# Dr. J. Milner Fothergill

**Published:** 1888-08

**Authors:** 


Dr. J. Milner Fothergill, one of the most celebrated and prolific medical writers, and a renowned therapeutist, died suddenly in London, June 28, 1888.

